# Supplementary material for: Affiliative behaviours regulate allostasis development and shape biobehavioural trajectories in horses
Source: Nat Commun. 2026 Jan 13;17:47. doi: 10.1038/s41467-025-66729-1 (PMC12800209; doi:10.1038/s41467-025-66729-1)
Supplement: Supplementary file 1 — Supplementary Information [file 41467_2025_66729_MOESM1_ESM.pdf]

Supplementary Information

Affiliative behaviors regulate allostasis development and shape biobehavioural trajectories in horse

Mathilde Valenchon[1,2$], Fabrice Reigner[3], Gaëlle Lefort[1], Hans Adriaensen[1,4], Amandine Gesbert[3], Philippe Barrière[3], Yvan Gaude[3], Frederic Elleboudt[1,4], Isabelle Lévy[5], Camille Ducluzeau[5], Joëlle Dupont[1], Anne-Lyse Lainé[1], Ivy Uszynski[6], Hugues Dardente[1], Cyril Poupon[6], Léa Lansade[1], Ludovic Calandreau[1], Matthieu Keller[1$] and David André Barrière[1$]

[1] CNRS, INRAE, Université de Tours, UMR Physiologie de la Reproduction et des Comportements, Nouzilly, France.

[2] Current address: Université Paris-Saclay, INRAE, AgroParisTech, UMR Modélisation Systémique Appliquée aux Ruminants, Palaiseau, France.

[3] INRAE, Unité Expérimentale de Physiologie Animale de l'Orfrasière, Centre de Recherche de Tours, Nouzilly, France.

[4] INRAE, Université de Tours, CHU de Tours, PIXANIM, Nouzilly, France.

[5] Veterinary Clinic of the Nouvetière, Sonzay, France.

[6] BAOBAB, NeuroSpin, Université Paris-Saclay, CNRS, CEA, Gif-sur-Yvette, France.

[$]Corresponding authors:

mathilde.valenchon@inrae.fr, matthieu.keller@inrae.fr, and david.barriere@cnrs.fr

Supplementary information – Behavioural assessment methods

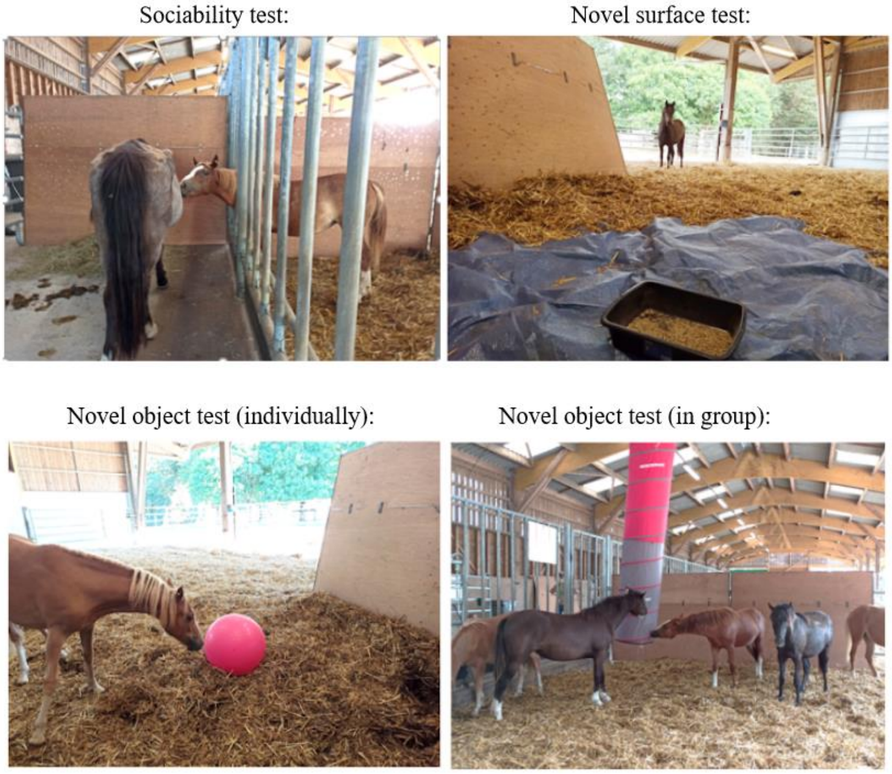

Supplementary Fig.1: Behavioural test to assess social behaviours in horse. Sociability test: A calm, non-reactive 23-year-old female Welsh pony was secured in the test area with forage and water to keep her still and close to the foals. A feed-through panel allowed foals to extend only their head and neck to initiate contact, while the pony could step back if needed; the time until each foal contacted the pony was recorded. Novel surface test: A plastic tarp with a centrally placed pellet bucket was set up out of sight before testing, and the time until each foal contacted the bucket was recorded. Novel object test (individual): A pink fitness ball was placed out of sight in the test area, and the time until each foal contacted the object was recorded. Novel object test (group): A foldable plastic tunnel, visible to all foals, was suspended in the middle of the testing area, and the time until each animal contacted the tunnel was recorded.

Supplementary information – Brain Imaging

The availability of *in vivo* population average templates and well-defined brain atlases is still a significant gamechanger for the use of the equine model in preclinical imaging[3–6]. Brain templates and atlas allow spatial normalisation and statistical analyses through group studies to compare the size/volume/fractions of pivotal structures based on microstructural imaging (anatomical MRI, diffusion MRI). Additionally, in the context of brain functional connectivity studies (fMRI) templates and cortical atlases allow the study of functional brain networks in different conditions[3–6]. To study equine brain using an MRI approach, only one resource was available[7]. This pioneer work proposes an *ex vivo,* unfixed, stereotaxic population template of the equine brain built from nine animals aged between 18 months and 30 years (7 males, 2 females) from different breeds (2 Quarter horse, 1 Mustang, 2 Standardbred, 3 Warmblood, 1 unspecified), three class of tissue segmentation maps for white matter (WM), gray matter (GM) and cerebrospinal fluid (CSF) and an atlas of 16 subcortical regions of interest[7]. However, this resource was characterised by significant limitations. First, all the animals used were adults at time of scanning and both gross and fine brain morphologies could be drastically different to our dataset in juvenile animals. Second, the cohort used to create both templates is sex-biased (~80% male *versus* 20% female) which is a limitation regarding the estimation of volumes in sexually dimorphic structures. Finally, the brain atlas proposed is largely incomplete with no cortical parcellation limiting, consequently the interest of such an atlas. In this context we built a new resource for the analysis of the equine brain, the Turone Equine Brain Template and Atlas (TEBTA), a new set of standardised MRI compatible templates and atlas in stereotaxic coordinates meant to support the analysis of multimodal MRI data of the equine brain.

Imaging acquisition and construction of the TEBTA

Anaesthesia

Imaging procedures have been realised in the PIXANIM imaging facilities, on the same site as animal' housing. Before being transported by van at the MRI platform (>5min course), animals were sedated to prevent stress (intramuscular injection of romifidine chlorhydrate 10mg/ml, single dose of 0.5mL Sedivet; Boehringer Ingelheim Vetmedica Inc, Beaucouze, France). Once at the MRI platform, anaesthesia was induced with a two steps process. First, an additional dose of romifidine hydrochloride 10mg/ml was injected intravenously as a premedication (0.14mg/kg, Sedivet). Five minutes afterward, ketamine hydrochloride 100mg/mL was injected intravenously to induce anaesthesia (2mg/kg, Imalgene, Boehringer Ingelheim Vetmedica Inc). Animal was then bedded in lateral recumbency and placed on a wheeled lift table. It was then placed on supine position onto the MRI table and maintained in this position with straps and memory foam mat and cushions to ensure stability, immobility and to prevent inadequate body pressure. A saphenous catheter and a urinary drain were then placed, and an ophthalmic ointment was applied on both eyes which were maintained closed during the whole imaging procedure to avoid ocular dryness. A veterinarian and an animal technician were constantly present near the animal to monitor the animal's respiratory and cardiac activities and general state, and to ensure constant and conformable anaesthesia. Additionally, a camera zooming on the head of the animal in the MRI tunnel enabled additional monitoring from the control room.

During the functional imaging procedure (15min approximately), guaifenesin 100 mg/mL (Myorelax, Dechra Veterinary Products SAS, Montigny-Le-Bretonneux, France) by perfusion and a ketamine/ xylazine was administered by additional intravenously injections to ensure anaesthesia. At the end of the functional imaging procedure, small injections of ketamine/Xylazine and, additionally, diazepam (Valium®) were made regularly at the discretion of the veterinarian. The mean duration of anaesthesia from the induction to the last injection was approximately of 3h and animals received a mean volume of 528.57±31.54mL of the perfusion mix (containing guaifenesin), plus 18.74±0.003mL of ketamine,

2.64±0.17mL of diazepam and 2.70±0.10mL of romifidine chlorhydrate through injections (initial sedation and induction included).

In vivo MRI acquisitions

MRI data have been acquired on 3 Tesla VERIO Siemens systems (Erlangen, Germany), using a two flexible coil (Siemens FLEX Large 4 elements) tied around the head and three MR acquisitions were performed on each animal. MR sequences have been optimized to 1/ be performed in a time compatible with anaesthesia (approximately 3h), 2/ reduce artefacts (folding, truncation, etc.) and 3/ optimise SNR. Regarding these specifications, the following sequences were used:

- for brain morphometry investigations, a three dimensional T_{1w} MPRAGE acquired in the coronal plane was used with the following parameters: Echo Time/Repetition Time=2.67 ms/2500 ms, Flip Angle=12°, Inversion Time=900 ms, Number of Excitation=3, Partial Fourier=1, Slice Thickness=1 mm, Slice Number=208, Field of View=256x256 mm, matrix=256x256, final resolution $1mm^3$.

- To investigate the brain microstructure, we developed a diffusion weighted MRI protocol based on a two dimensional T_{2w} spin-echo sequence acquired in the axial plane, over 3 different shells optimized for Neurite Orientation Dispersion and Density Imaging (NODDI) model using the following parameters: Shell 1: b=300 s/mm^2, 6 directions; Shell 2: b=700 s/mm^2, 30 directions, and Shell 3: b=2000 s/mm^2, 64 directions. The fixed parameters are Echo Time/Repetition Time=109ms/11.5s, Flip Angle=90°, Number of Excitation=1, Partial Fourier=0.75, Slice Thickness=2.4 mm, Slice Number=57, Field of View =256x256 mm, matrix=128x128, final resolution 2x2x2.4 mm^3, one b=0 per shell). Sequences have been acquired in different reading phases (left-right and right-left) for distortion corrections.

-To investigate brain functioning a T_{2w} spin-echo-planar imaging (SE-EPI) sequence acquired in the axial plane and left/right reading phase was used with the following parameters: Echo Time/Repetition Time=24 ms/3.97 s, Flip Angle=90°, Number of Excitation=1, Partial Fourier=1, Slice Thickness=3.3 mm, Slice Number=40, Field of View=220x220 mm, matrix=110x110, final resolution 2x2x3.3 mm^3, Number of repetition=250. Additionally, two similar sequences in different reading phases (left-right and right-left) of ten volumes each were acquired for distortion corrections.

Once the imaging procedure finished, the animal was immediately transported back to a recovery stable to wake up progressively. Hydration (3 mL of Ringer minimum), flunixine meglumine (single dose of 3.5mL, Antalzen, 50 mg/mL, laboratorios calier, Barcelona, Spain) and of acefylline heptaminol (a single dose of 25mL of Vetecardiol, Intervet MSD Santé animale, Beaucouzé, France) were systematically administered. Veterinarian and at least two animal technicians were constantly present near the animal to monitor its recovery until it was able to walk steadily (approximately 1h40 for recovery). Then, the animal stayed in the recovery stable at least 6 hours after he stood-up for surveillance with another animal (the mother or another foal depending on the group) to avoid social isolation stress and finally joined back their housing. A blood sample was taken 48h after anaesthesia and sent to the veterinary office to check the absence/presence of myositis. Only one case of myositis has been detected afterward nevertheless the animal fully recovered within a week. DICOM data from the scanner were converted to NIFTI format and organized as standardized data sets according to the Brain Imaging Data Structure (BIDS) using BIDScoin and are downloadable on Zenodo.

TEBTA template creation

T_{1w} MPRAGE data acquired for each animal (23 scans) were noise and signal bias corrected using Ginkgo and N4BiasFieldCorrection respectively and coregistered to the Johnson *et al* template[7] using antsRegistrationSyNQuick. Coregistrated data were used to segment each brain using SPM and the GM, WM and CSF priors provided by Johnson *et al* to create the GM, WM and CSF probabilistic maps of each subject. Denoised and signal bias corrected images were used in parallel to create a study specific T_{1w} template, using modelbuild, an optimized pipeline using antsMultivariateTemplateConstruction2,

an unbiased template building method developed in ANTs package. This method has been integrated to our local cluster (ISLANDe Facilities) and requires qbatch and slurn. For computation, our cluster comprises 240 physical cores, each associated with 9.6GB of memory (2.3TB of memory in total), with 90TB of long-term storage and 72TB of high-performance storage. Once both linear and non-linear (flowfield maps) transformations were calculated for each animal, we compiled all the transformations calculated for each image and applied them once to denoised and signal bias corrected images using antsApplyTransforms to limit interpolation effects. Resulting images have been used to create the TEBTA template by calculating the mean image of each normalized T_{1w} using Ginkgo. Probabilistic maps (GM, WM, CSF) have been normalized using the linear and non-linear transformation provided by modelbuild using the antsApplyTransforms command and the TEBTA GM, WM and CSF priors have been created by calculating the mean image of each normalized map using Ginkgo. Both templates and priors will be used for spatial normalization and segmentation for VBM analysis (see *Voxel Based Morphometry Analysis* section of main article).

TEBTA atlas creation

For the creation of the TEBTA atlas, the equine brain atlas provided by Johnson *et al* is used as a starting point. Firstly the Johnson' template is linearly and nonlinearly coregistrated within the TEBTA space using antsRegistrationSyNQuick. Then, both linear and non-linear transformations were applied to each ROI of the Johnson' atlas using antsApplyTransforms. Each normalized ROI has been visually inspected to check boundaries and accuracy of registration. Then some regions of interest (ROIs) such like *Corpus Callosum*, *arbor vitae*, and ventricular systems have been updated/added using WM and CSF priors for a best fit to these ROIs to the TEBTA template. Eventually, additional subcortical ROIs such as septum, preoptic hypothalamic area, nucleus accumbens, striatum, etc. have been drawn and delimited manually using fsleyes and itksnap using both GM and WM priors maps to determine the boundaries of each new ROI.

To propose a valuable parcellation of the equine cortex, we implemented the methods previously used by Garin *et al* for the establishment of a functional atlas in lemur mouse[8]. Briefly, a multi-animal dictionary learning statistical analysis was performed with Nilearn (random_state = 0) on preprocessed rsfMR images (see *Functional Imaging Analysis* section of main article)[9]. A mask excluding the WM, CSF and subcortical areas was used to restrict the dictionary learning analysis to cortical functional data. During a pilot investigation, several analyses were performed using 30, 35, 40, 45, 50, 60, 90 and 120 sparse components. The study based on 60 sparse components was selected for the final analysis as it highlighted bilateral regions that matched well to anatomy or unilateral regions located to the sagittal midline (cingulate-related regions). Each bilateral component was split into two unilateral regions and labelled left or right. Regions smaller than 5 mm^3 were excluded. This led to a 3D functional atlas composed by a mosaic of 55 local functional regions that were named using ITK-SNAP. The name of each ROI was defined using the names of brain structures reported by Schmidt *et al*[10], the AAL2 human brain atlas but also using their structural connectivity.

TEBTA fiber atlas creation: Structural connectivity of the equine brain

Cortical ROIs resulting from the dictionary learning segmentation of the equine cortex were used to identify the largest bundle tracts of white matter to help for the identification of ROI based on their structural connectivity. For the construction of the fibre tractogram, we used the analytical Q-ball reconstruction model[11] since this model relies on the decomposition of the diffusion-weighted signal into a modified spherical harmonics basis. We firstly computed fields of the orientation distribution functions (ODFs) stemming from the analytical Q-ball model including the fractional anisotropy map (FA), the mean diffusivity maps (MD), and the colour-encoded direction (CED) map. The analytical Q-ball model reconstruction was applied using spherical harmonics order 8 and a regularization factor λ = 0.006. Then, a streamline regularized deterministic (SRD) tractography algorithm[11] was applied to the

whole sample using the ODFs maps of aQBI previously computed, with the following parameters: 8 seeds per voxel, forward step 400 µm, maximum solid aperture angle 30°, minimum/maximum fibres length 10/300 mm. In the next step, tractogram were normalized to the TEBTA template using the previously calculated affine and diffeomorphic transformations (see *Diffusion Imaging Analysis* section of main article). Tractograms of each animal have been reduced by a random selection of 5% of total fibre count composing the tractogram and all the tractograms (n=23) were fused together and symmetrized to create a population-based tractogram. From this tractogram, we selected the large bundles (length>150mm) using a 2 steps approach. 1/ Bundles selection: in this step, bundles between each cortical ROIs have been selected by a ROI-to-ROI selection, labelled and merged. Then bundles between thalamus and each cortical ROI were selected as well as bundles between cerebellum and each cortical ROIs. With this selection, we expected to find long cortico-cortical pathways (i.e. cingulum), the thalamic projections such as the somatosensorial tract to identify the somatosensory areas and the cerebellocortical tract to identify the motor areas. To eliminate non-relevant fibres previously selected within the bundle tract we filtered them by length (>150mm) and by tortuosity (<2σ of mean of tortuosity). 2/ building of fibre atlas: here all the bundles selected have been visually inspected and 7 large bundle tracts (cingulum, corticospinal tract, anterior/posterior/inferior thalamic radiations, inferior longitudinal tracts and cerebellocortical tract) have been identified on basis of morphology, position within the brain, location and by comparison of fibre atlases available in humans and NHP. Areas connected to these tracts have been named according to their structural connectivity, position and literature[10]. All tractogram calculations and manipulation have been done by using Ginkgo toolbox developed by the Ginkgo team headed by C. Poupon. TEBTA resources are freely available on Zenodo.

Brain imaging quality control

Equine brain template creation and comparison.

The TEBTA was built using the methods developed by the ANTs package. Comparison of signal to noise ratio (SNR) and contrast to noise ratio (CNR) between Johnson template and TEBTA are summarized in **Table S1**.

	Johnson et al 2019	TEBTA
**Contrast**	T1w	T1w
**Number of subjects**	9	23
**Sex Ratio**	0,29	1,09
**Sequence**	MPRAGE	MPRAGE
**Repetition time (s)**	7.36	2.5
**Echo Time (ms)**	3.47	2.67
**Inversion time (ms)**	425	900
**Average**	1	3
**Resolution (mm³)**	1	0.8
**SNR**	2.46	8.03
**CNR**	3.49	1.72
**Number of Region of interest**	16	115

Supplementary Table 1. Comparison of Johnson and Turone equine brain templates characteristics.

SNR is higher for the TEBTA template (Jonhson=2.46 vs. TEBTA=8.03; +330%) while CNR is higher for the Johnson' template (Jonhson=3.49 vs. TEBTA=1.72; -50%). Additionally, brain segmentation looks different between both resources. When the Johnson' segmentation maps (FSL-FAST) seem to classify massively brain voxels in the white matter compartment (WM) rather than within grey matter compartment (GM), the TEBTA classification (Atropos) enlarged the cortical band and change voxels from WM to GM within caudate and mesencephalon (Supplementary Fig.2a-b, blue arrows). Additionally, the shape of ventricles looks different and are enlarged in TEBTA (Supplementary Fig.2a-b, orange arrows) probably because T_{1w} data have been acquired *in vivo* for TEBTA versus *ex vivo* in

Johnson' resources. Finally, some unexplained and extracephalic voxels classified as cerebrospinal fluid (CSF) are observed within the Johnson' CSF prior but not in TEBTA CSF prior maps (Supplementary Fig.2a-b, red arrows).

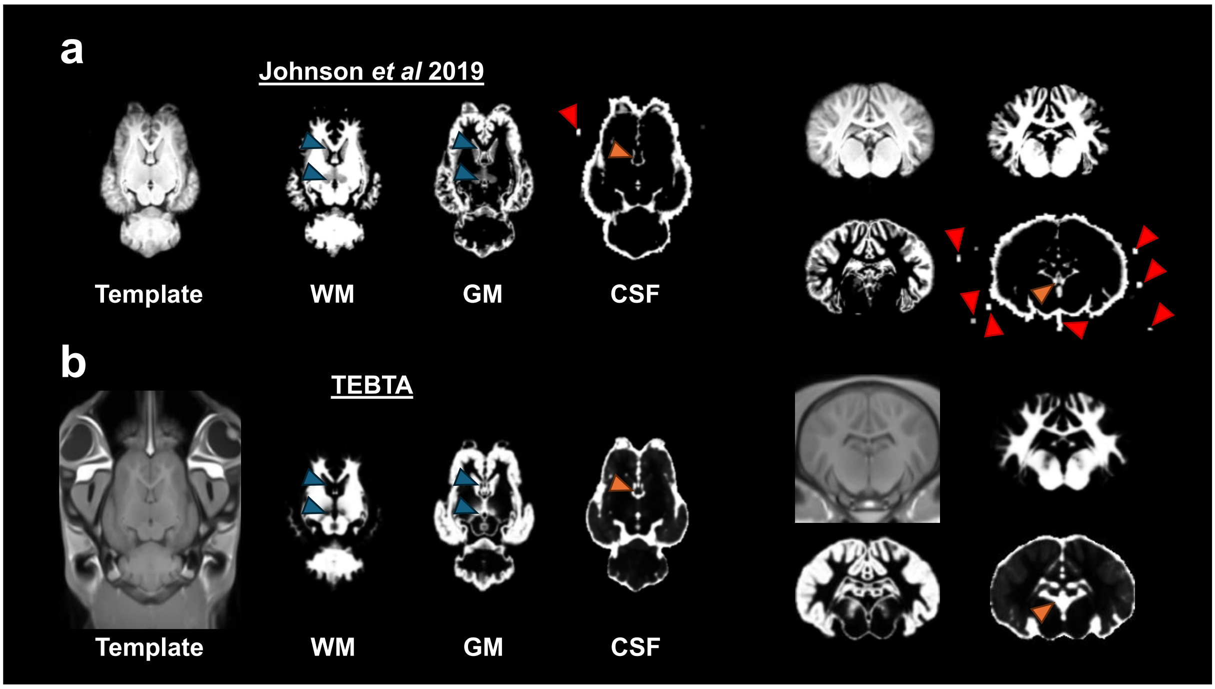

Supplementary Fig.2: The Turone Equine Brain Template & Atlas. Sagittal and axial brain slices of the Johnson **(a)** and TEBTA **(b)** equine templates and their associated tissue probability maps of grey matter (GM), white matter (WM) and cerebrospinal fluid (CSF). All the images have been linearly co-registered within the same space for comparison. Blue arrows show the differences of classification between GM and WM through both resources. Oranges arrows show the differences of classification between GM/WM and CSF through both resources. Red arrows show extracephalic voxels misclassified as CSF.

Controls and Quality Assessments.

As this study is pioneering in the field, we provide in this section a random selection of raw data obtained with MRI sequences used in our work.

Functional imaging

Here, we used Spin-Echo EPI (SE) for resting-state fMRI. The main reason for using a SE sequence for brain imaging in foal is the sensibility of GE for susceptibility artifacts. Indeed, SE sequences are particularly beneficial in brain regions near air-tissue or bone-tissue interfaces, where geometric distortions are problematic and regarding the size of frontal sinus, guttural pouch and eustachian tubes in these animals, such artefacts could be dramatic for the subsequent functional MRI analysis (Supplementary Fig.3). Sequences proposed here to study functional connectivity, preserved BOLD signal within frontal areas, amygdala, hypothalamus, etc (Supplementary Fig.4) with a good SNR (44.60 ± 4.14) and tSNR (23.39 ± 3.96) (Supplementary Table 2).

Animal	Functional data		Diffusion Data Shell 1			Diffusion Data Shell 2			Diffusion Data Shell 3		
	SNR	tSNR	SNR b0	SNR b = 300 m/s^2	tSNR b = 300 m/s^2	SNR b0	SNR b = 700 m/s^2	tSNR b = 700 m/s^2	SNR b0	SNR b = 2000 m/s^2	tSNR b = 2000 m/s^2
**sub-01**	42,92	16,72	26,14	18,22	12,78	26,35	12,69	7,78	26,49	5,49	3,54
**sub-02**	45,07	15,30	22,52	15,20	11,97	22,91	10,34	7,58	23,16	4,44	2,77
**sub-03**	49,21	19,40	24,67	16,58	11,95	24,77	11,20	7,63	25,29	4,85	3,45
**sub-04**	46,69	20,59	25,28	16,96	13,92	25,39	11,40	8,64	25,84	4,82	3,72
**sub-05**	41,56	24,48	23,53	16,36	11,40	23,93	11,75	7,61	24,70	5,27	3,48
**sub-06**	34,08	25,97	35,61	24,94	14,21	35,87	17,83	9,28	36,93	7,73	3,99
**sub-07**	40,05	19,43	25,19	17,93	10,50	25,66	12,86	7,27	26,15	5,69	3,27
**sub-08**	50,09	28,44	30,66	20,83	14,68	30,89	14,11	9,38	31,61	5,92	4,01
**sub-09**	38,79	22,91	25,07	19,41	13,50	25,13	14,75	8,50	25,25	6,55	3,67
**sub-10**	43,28	24,27	28,81	20,18	13,08	29,17	13,87	8,43	29,63	5,85	3,77
**sub-11**	47,92	27,85	26,04	19,12	14,74	26,42	13,62	9,42	27,11	5,99	4,08
**sub-12**	46,76	19,34	27,37	19,20	13,52	27,73	13,14	8,53	28,24	5,54	3,69
**sub-13**	48,17	26,88	23,62	17,14	13,11	23,72	11,98	8,21	24,27	5,19	3,65
**sub-14**	44,78	28,80	25,77	18,00	13,52	25,76	12,59	8,68	26,45	5,40	3,76
**sub-15**	45,48	26,15	21,39	14,89	11,51	21,81	10,76	7,65	22,92	4,95	3,55
**sub-16**	39,72	21,83	21,94	15,36	11,41	22,18	10,80	7,33	23,24	4,91	3,33
**sub-17**	39,34	19,89	25,17	18,00	13,29	25,21	12,35	8,07	25,95	5,28	3,44
**sub-19**	42,96	27,19	25,41	17,79	12,69	25,92	12,24	6,06	24,33	5,24	3,08
**sub-20**	47,41	19,83	29,95	19,77	13,40	30,44	13,30	8,62	30,48	5,53	3,74
**sub-21**	50,22	22,66	24,03	17,08	13,21	24,24	12,22	8,62	24,83	5,44	3,84
**sub-22**	47,42	27,84	22,93	15,32	12,33	23,09	10,74	8,08	23,77	4,77	3,63
**sub-23**	46,67	26,13	23,67	15,84	12,18	24,06	11,24	8,06	24,87	4,99	3,64
**sub-24**	47,32	26,03	21,97	15,41	12,27	22,17	10,83	8,03	22,57	4,77	3,58
**Mean**	**44,60**	**23,39**	**25,51**	**17,81**	**12,83**	**25,77**	**12,46**	**8,15**	**26,27**	**5,42**	**3,59**
**SD**	**4,14**	**3,96**	**3,27**	**2,32**	**1,09**	**3,29**	**1,68**	**0,76**	**3,32**	**0,70**	**0,30**

Supplementary Table 2. Calculation of SNR and tSNR for both functional and diffusion data for each animal included in the present study.

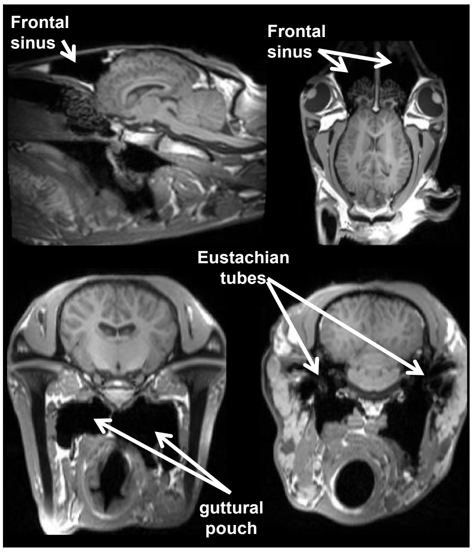

Supplementary Fig.3: Raw anatomical T_{1w} images of the foal' head centred on the brain. Images show sagittal (top left), axial (top right) and coronal (bottom left and right) sections showing how the horse brain is surrounded by large cavities such as frontal sinus, guttural pouch and eustachian tubes connecting guttural pouch.

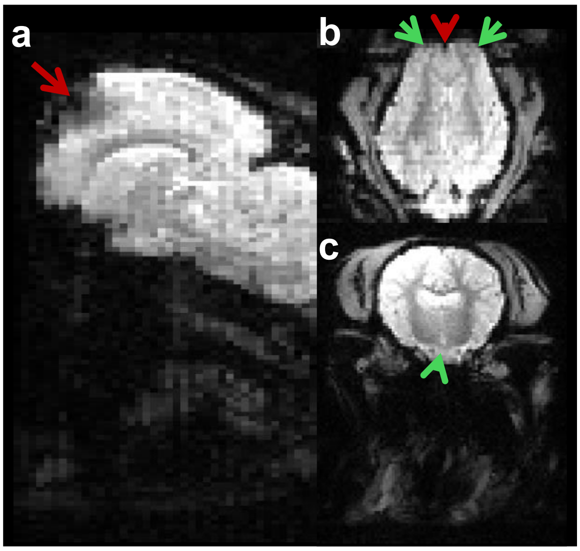

Supplementary Fig.4: Raw functional images (spin echo). Images show sagittal **(a)**, axial **(b)** and coronal **(c)** sections showing raw images for quality assessment. Using SE sequence, a small artefact is visible within the region close to frontal sinus but only at the interhemispheric level (**a**, red arrow). Signal in frontal regions is preserved (**b**, green arrows). No artefact is observed in structures close to both guttural pouch and eustachian tubes (hypothalamus in **c**, green arrow).

From the obtained functional data, we evaluate the motions of animals during functional acquisitions. As previously described, animals were firmly maintained with straps and memory foam mat during imaging procedure and anesthetized with a ketamine/xylazine cocktail to ensure anaesthesia. Translations and rotations during functional acquisition were estimated using SPM and analysis showed that minimal translations and rotations occurred during functional MRI using our anaesthesia procedure (translations < 1mm, Supplementary Fig.5a ; rotations < 0.3°, Supplementary Fig.5b).

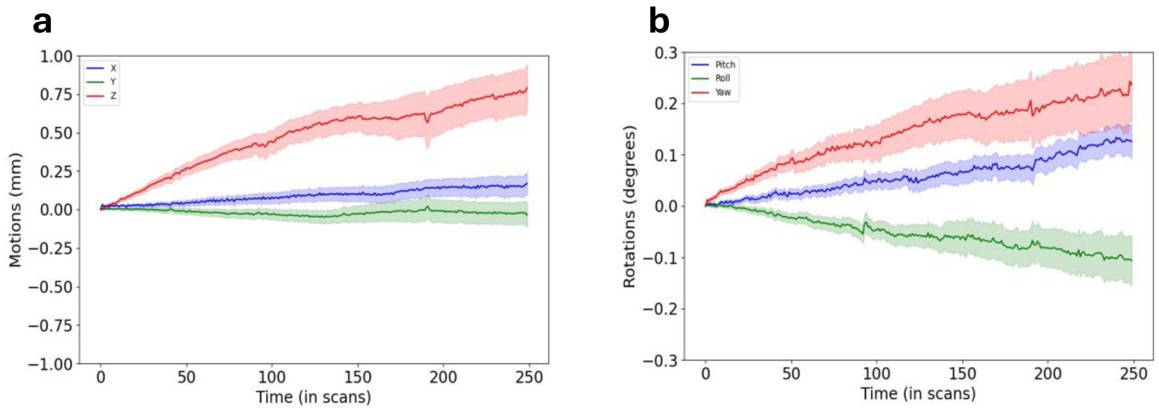

Supplementary Fig.5: Motions parameters recorded during acquisition. (a) translations, **(b)** rotations. Data are expressed as mean ± SD.

Large scale networks identification.

rs-fMRI data were pre-processed as previously described[3,12,13]. Briefly, EPI images were corrected for slice timing (slicetimer), motions (antsMotionCorr) and susceptibility distribution (topup). Then mean of EPI data for each animal were calculated and resulting images were used to calculate a functional template using by modelbuild. Resulting template was then normalized to the TEBTA template using antsRegistrationSyNQuick. Then images were detrended, low- and high-pass filtered (0.01Hz – 0.001Hz) and the effect of the six previously calculated motion parameters including translations and rotations, both WM and CSF and global signals, were removed from data through linear regression using Nilearn. Finally, an Independent Component Analysis (ICA) was run for each subject to detect the remaining noise and artifact signals using the melodic tool (Multivariate Exploratory Linear Optimized Decomposition into Independent Components) of FSL. This process decomposes the fMRI signal into different time courses, which are in turn translated into spatial maps. The process thus quantifies the time-course association of each voxel, while optimizing the spatial statistical independence of the maps. The resulting components were visually inspected, and the ones identified as artefacts regressed from the original data [3,12,13].

Form this cleaned dataset, we feed the Dictionary learning (or sparse coding) method proposed in Nilearn to identify the large scale networks. Dictionary learning is a representative learning method aiming at finding a sparse representation of the input data as a linear combination of basic elements called atoms. The identification of these atoms composing the dictionary relies on a sparsity principle: maximally sparse representations of the dataset are sought for. Atoms are not required to be orthogonal[8,9,14]. To identify large scale networks, 6 components were prescribed to the dictionary learning function (smoothing = None, mask, n_components = 6, memory = "nilearn_cache", memory_level = 2, alpha = 5, standardize = False, detrend = None, random_state = 0, n_epochs = 1). We formally identified 4 on 6 networks using information from the literature (Supplementary Fig.6):

1/ the occipito-parietal network involving the occipital and parietal posterior regions, temporal middle/inferior, and cingulum posterior/precuneus cortices. This component evokes the visual network reported in humans[15,16].

2/ the default mode network (DMN) identified from its three core clusters, the medial frontal cortex, precuneus region and posterior cingulate/retrosplenial cortices, and angular cortices[15–17].

3/ the mid-cingulate network encompassing the mid cingulate and bilateral parietal cortices which should be consider as a part of the dorsal attentional or the sensorimotor network[15–17].

4/ the frontal network embedding the frontal anterior medial and lateral regions, the anterior cingulum cortices, and the insula which could be referred as the attentional network.

The two last components segregated by the dictionary learning methods are lateralized either on left side or on right-side and should be interpreted as both executive networks left and right (data not shown, see the dictionary_learning_resting_state_EquineBrain.nii.gz Nifti image in attached).

However, the precise identification of large network in equidae is limited by the small of animals included in our study and additional validations are needed for each network. For this reason, we focused our interest on the default mode network which is on particular interest in the context of our study (see main article and [18,19]).

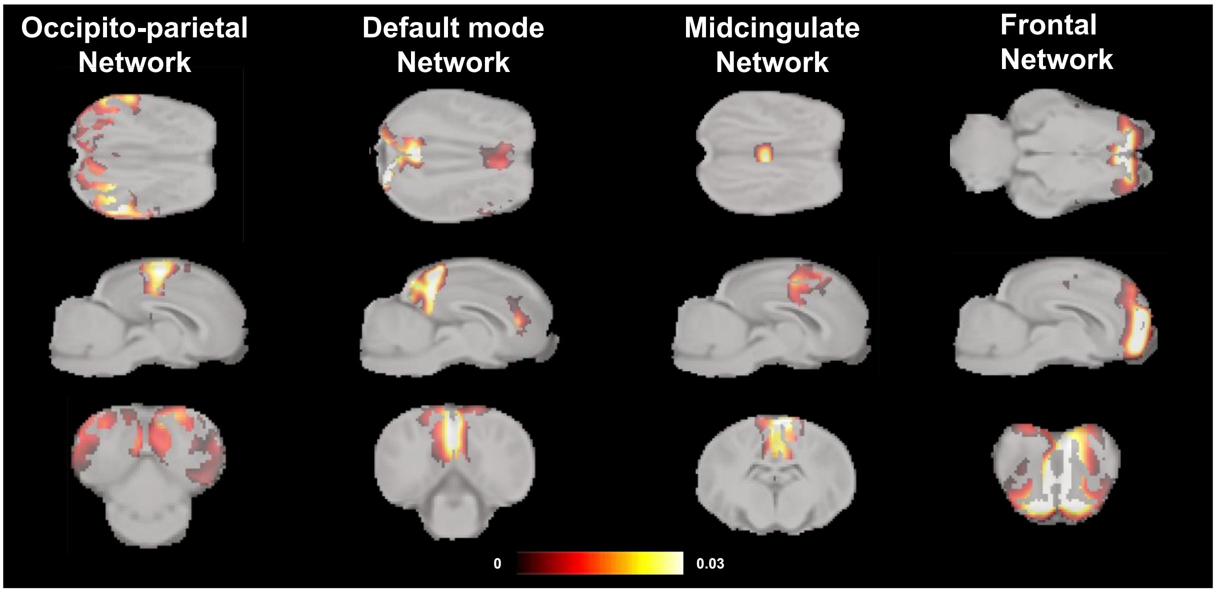

Supplementary Fig.6: Large scale networks of equidae. Using a dictionary learning approach we identified the occipito-parietal network, the default mode network, the midcingulate network and the frontal network in foals**.**

The default mode network of equidae.

To validate the identification of the DMN defined by the dictionary learning method, we used a seed-based approach using the ROI defined as the *cuneus/precuneus* (Cu/PrCu) within the TEBTA atlas. This region is chosen based on the previous observations that this posterior region belong to the DMN and should be functionally connected to the frontal regions of the brain but also with both left and right angular cortex through the DMN network[15–17]. Here, we thus investigated the correlation between the temporal fluctuation of the BOLD signal within the Cu/PrCu seed region with the temporal fluctuation of BOLD signal with the rest of the brain. This correlation analysis helps to identify areas of the brain that show similar patterns of activity to the Cu/PrCu region and allowed to validate our observations of the DMN of equidae and study the functionality of the DMN of our both groups. To do so, the time series data from the Cu/PrCu were correlated with the time series data from all other voxels in the brain through a multiple regression analysis in SPM, a statistical method employing the general linear model (GLM) framework used to model the relationship between brain activity and variables. At the first level (individual), the design matrix includes 21 regressors whose the first one was the extracted time-series of the temporal fluctuation of the BOLD signal within the Cu/PrCu, and the rest used as confound variables namely the 6 motions parameters (rotation, translations), the WM and CSF and global signals and the 10 major components identified as noise and artifact signals by the previously by melodic. For each individual, the Cu/PrCu BOLD fluctuation positively correlate with both left and right angular cortex and frontal regions and negatively correlate with a midcingulate region which could be associated with the somatosensory network (Supplementary Fig.7)[19,20].

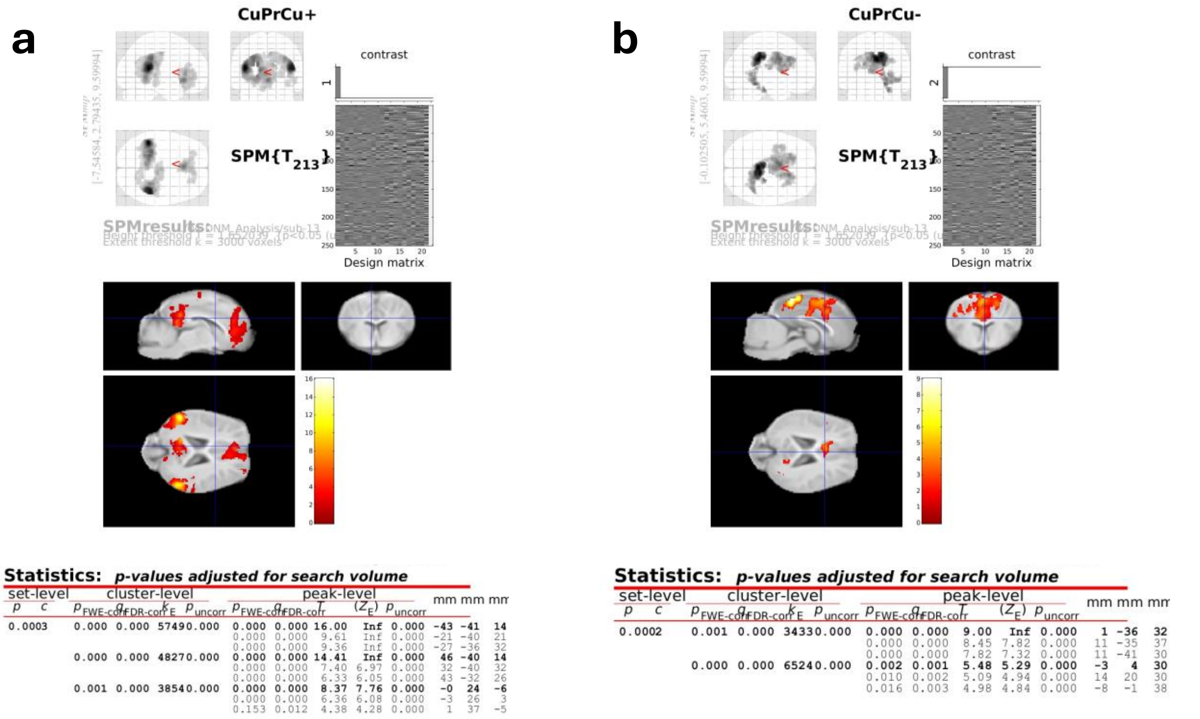

Supplementary Fig.7: Results of the 1st level of SPM multivariate analysis for one individual. (a) Positives correlations between Cu/PrCU and the rest of the brain, **(b)** negatives correlations. Data were obtained from SPM multiple regression analysis using a voxel-level threshold p < 0.05, t-threshold = 1.65, two-tailed, FDR-corrected, voxel threshold = 300.

Then we performed the 2nd level of analysis to highlight the DMN network at the group level using T-maps obtained from the 1st level. Data thresholded at two different levels (Supplementary Fig.8a, $p < 0.05$, t-threshold = 3.46, two-tailed, FDR-corrected, voxel threshold = 3000 and Supplementary Fig.8b, $p < 0.001$, t-threshold = 7.054, two-tailed, FDR-corrected, voxel threshold = 3000) show a high correlation between angular gyrus (aG), anterior cingulate cortex (aCC) and cuneus/precuneus (Cu/PrCu) as canonically reported for the DNM in numerous species including humans[15–17]. Additionally, anticorrelated voxels were found bilaterally within the somatosensorial cortical area (S1), which is consistent with previous descriptions of DMN and DMN-like networks in humans, mice and rats.

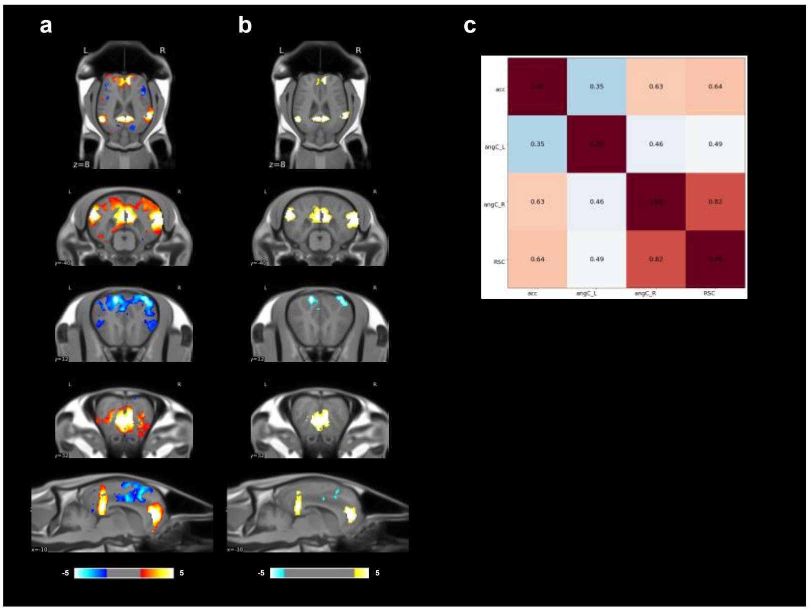

Supplementary Fig 8: Default mode network of equidae. (a) data thresholded at p < 0.05, t-threshold = 3.46, two-tailed, FDR-corrected, voxel threshold = 3000. **(b)** data thresholded at p < 0.001, t-threshold = 7.054, two-tailed, FDR-corrected, voxel threshold = 3000. **(c)** correlation matrix of the canonical brain structures embedded in the DMN. Abbreviations: angG L = angular gyrus left; angG R = angular gyrus right; aCC = anterior cingulate cortex; Cu/PrCu = cuneus/precuneus.

Equine brain atlas creation.

A brain atlas is a cartography of the brain which is mandatory for the identification of brain territories involved in a biological or pathological process. The Johnson' atlas is composed by a mosaic of 16 subcortical regions of interest (data not shown, see[7]) but no cortical regions have been segmented. To propose an accurate cortical parcellation of the equine brain, we used functional MRI data acquired at rest and use a multi-animal dictionary learning statistical approach to emulate a functional cortical atlas of equidae. Using this approach, we identified 55 functional regions in the equine cortex. Among these regions, 9 were located on the sagittal midline and associated as frontal regions or cingulate cortex parts (anterior cingulate, anteromedial cingulate, cuneus/precuneus, frontal anteromedial, orbitofrontal, posterior cingulate, posteromedial cingulate, retrosplenial, superior frontal). The other regions were found bilaterally with an acceptable similar shape and location between both hemispheres. Naming of these functional regions was based on available literature20 and human AAL2. Then we conserved and normalised the 16 original subcortical labels from the Johnson atlas to the TEBTA. After visual inspection and manual correction, we added 48 subcortical regions (i.e. septum, preoptic hypothalamic area, nucleus accumbens, striatum, etc.) which have been drawn and delimited using GM and WM priors' maps (Fig.2a in the main article). Both cortical and subcortical labelling were finally merged to emulate a complete brain atlas composed by a mosaic of 119 ROIs (Fig.2b in the main article). Using this atlas and tractograms calculated from the diffusion weighted imaging, we inferred a fibre atlas of the equine brain which identifies the largest bundles tracts of white matter for the identification of the ROI based on their structural connectivity (Fig.2c in the main article). We identified 7 large bundle tracts: the cingulum which permitted to identify cingulate cortices (anterior, medial, posterior) and to decipher frontal regions[21], the corticospinal tract for the identification of the somatosensory regions[22], the anterior thalamic radiations for the identification of the prefrontal region[23], the posterior thalamic radiations for the identification of the occipital lobe[23], the inferior thalamic radiations for the identification of the insula[23], the inferior longitudinal tract one of the major occipitotemporal used for the identification of occipital areas[24], and the cerebellocortical tract for the identification of motor regions[25]. Finally, ROI naming was terminated using neuroanatomical information found in literature and homologous unnamed regions were identified using ALL2 human brain atlas.

Diffusion imaging

Here, we used three T_{2w} SE sequences with different b-values and directions (Shell 1: b=300 s/mm^2, 6 directions; Shell 2: b=700 s/mm^2, 30 directions, and Shell 3: b=2000 s/mm^2, 64 directions) for diffusion imaging. Parameters used for this section of the study allowed to get a comfortable SNR and tSNR (Supplementary Table 2) suitable for the calculation of the main diffusion parameters to build both fractional anisotropy (FA) and fiber orientation maps for each foal (Supplementary Fig.9a) and for the creation of both the FA and fiber orientation template of equidae (Supplementary Fig.9b). Quality of those maps are important for the estimation of tractograms used for the identification of brain territories (see Fig.2 in the main article) as well as to evaluate the robustness of the raw data for NODDI analysis.

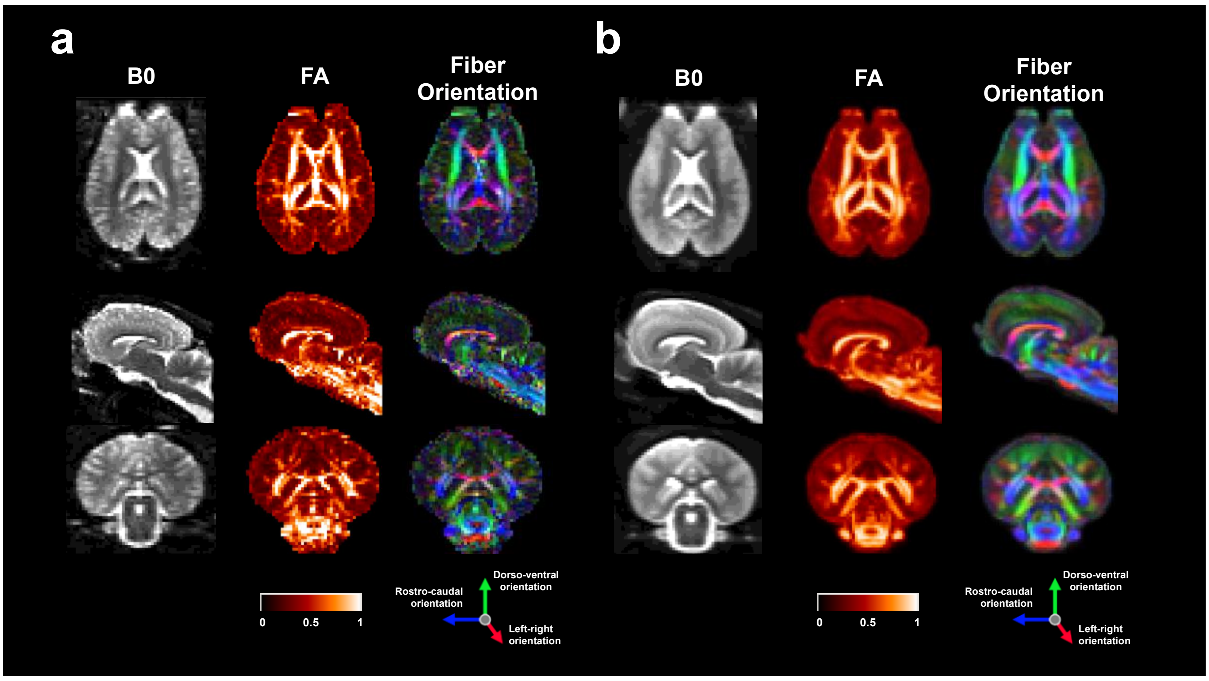

Supplementary Fig 9: Diffusion imaging in equidae. Maps of B0, FA and RGB-encoded principal direction computed using our three-shell dataset, showing axial (top), sagittal (middle) and axial (bottom) brain sections at the individual level **(a)** and population level **(b)**.

Supplementary Methods 2: physiological assessment

Assessment of endocrine and metabolic phycological markers

Plasma metabolites and hormones (insulin and IGF-1) assays

The concentrations of plasma total cholesterol (Chol) and triglycerides (TGs) were measured by using the respective assays (References 80106 and 80019, Biolabo, Maizy, France). Plasma glucose levels were determined by using the GAGO-20 kit (Sigma Aldrich, Saint-Quentin Fallavier, France). Plasma insulin and IGF-1 were determined by using the equine insulin and IGF-1 enzyme-linked immunosorbent (ELISA) assays (MBS044785 and MBS017382; MyBioSource Inc, San Diego, CA) according to manufacturer's protocol. For all the measurements of these plasma metabolites and hormones, the inter-assay and intra-assay coefficients of variation were <8%.

Oxytocin assay

Oxytocin concentrations were determined by Enzyme ImmunoAssay (Enzo Life Sciences, ADI-901-153) after solid-phase extraction using Sep-Pack C18 cartridges (Waters, WAT054945). 1000µl of plasma samples were extracted and reconstituted in 230µL assay buffer. Measurements were performed in duplicate. The intra-assay coefficient of variation was 5.8 % at 40pg/mL and the assay sensitivity was 7.8pg/mL.

Cortisol assays

Cortisol concentrations were determined using competitive enzyme immunoassay after steroid extraction with a solvent (ethyl acetate/cyclohexane). Duplicate analyses were performed on each sample. The intra-assay coefficients of variation were 2.8% and 2.7% at 15ng/mL and 60ng/mL respectively. The assay sensitivity was 2ng/mL.

Baseline comparison

Regarding metabolite and hormone concentrations, we did not find any significant differences between the “maternal presence” and “maternal separation” groups one month before maternal separation (Mann-Whitney test, p value set < 0.05, Supplementary Table 3, Supplementary Fig.10).

Value Column	U	U (standardized)	Expected Value	Variance (U)	p value (two_tailed)	alpha
Triglycerides (g/L)	62.0	-0,577	72	300	0,583	0,05
Cholesterol (g/L)	61.0	-0,635	72	300	0,544	0,05
Uric Acid (Âµg/L)	72.5	0,029	72	300	1,000	0,05
Insulin (mIU/L)	54.0	-1,039	72	300	0,312	0,05
IGF-1 (ng/L)	66.0	-0,346	72	300	0,751	0,05
Basal Cortisol (ng/mL)	98.5	1,530	72	300	0,133	0,05

Supplementary Table 3. Summary table of statistical analysis for non-weaned *versus* weaned group comparison of plasmatic concentrations of triglycerides, cholesterol, uric acid, insulin, IGF-1, and basal cortisol one month before maternal separation (Mann-Whitney test).

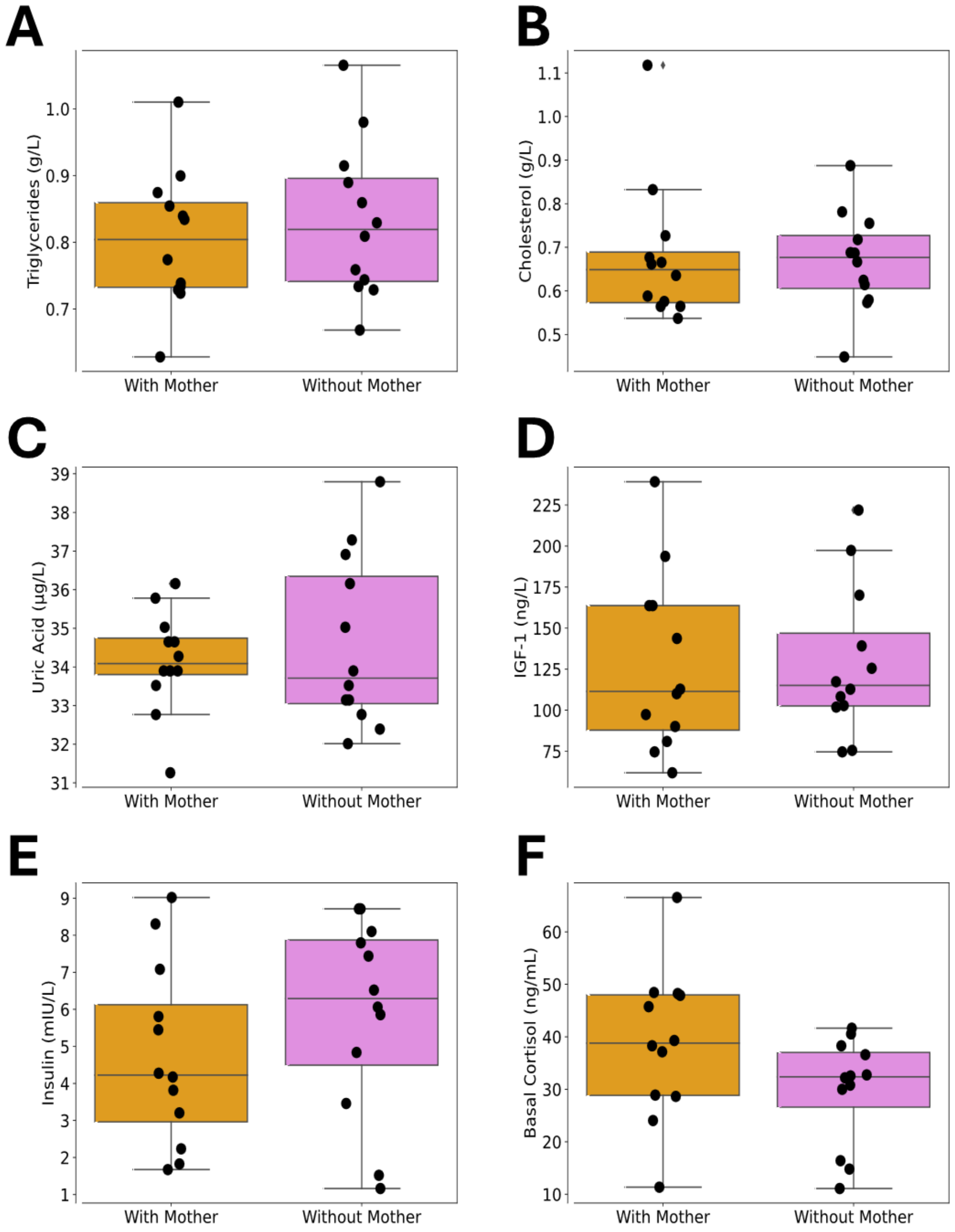

Supplementary Fig.10: Basal levels of physiological markers. Plasmatic concentration of triglycerides **(a)**, cholesterol **(b)**, uric acid **(c)**, IGF-1 **(d)**, insulin **(e)** and basal cortisol **(f)**, 1 month before maternal separation in animals eventually included in "*without mother*" (weaned) or in "*with mother*" (non-weaned) group.

References

1. Lansade, L., Bouissou, M.-F. & Erhard, H. W. Fearfulness in horses: A temperament trait stable across time and situations. *Appl. Anim. Behav. Sci.* **115**, 182–200 (2008).
2. Lansade, L., Bouissou, M.-F. & Erhard, H. W. Reactivity to isolation and association with conspecifics: A temperament trait stable across time and situations. *Appl. Anim. Behav. Sci.* **109**, 355–373 (2008).
3. Barrière, D. A. *et al.* The SIGMA rat brain templates and atlases for multimodal MRI data analysis and visualization. *Nat. Commun.* **10**, 5699 (2019).
4. Evans, A. C., Janke, A. L., Collins, D. L. & Baillet, S. Brain templates and atlases. *NeuroImage* **62**, 911–922 (2012).
5. Mars, R. B. & Palomero-Gallagher, N. Towards multi-modal, multi-species brain atlases: part one. *Brain Struct. Funct.* **228**, 1041–1044 (2023).
6. Mars, R. B. & Palomero-Gallagher, N. Towards multi-modal, multi-species brain atlases: part two. *Brain Struct. Funct.* (2024) doi:10.1007/s00429-024-02858-5.
7. Johnson, P. J. *et al.* Equine Stereotaxtic Population Average Brain Atlas With Neuroanatomic Correlation. *Front. Neuroanat.* **13**, (2019).
8. Garin, C. M. *et al.* Resting state functional atlas and cerebral networks in mouse lemur primates at 11.7 Tesla. *NeuroImage* **226**, 117589 (2021).
9. Mensch, A., Mairal, J., Thirion, B. & Varoquaux, G. Dictionary Learning for Massive Matrix Factorization. in *Proceedings of The 33rd International Conference on Machine Learning* 1737–1746 (PMLR, 2016).
10. Schmidt, M. J., Knemeyer, C. & Heinsen, H. Neuroanatomy of the equine brain as revealed by high-field (3Tesla) magnetic-resonance-imaging. *PLOS ONE* **14**, e0213814 (2019).
11. Perrin, M. *et al.* Fiber tracking in q-ball fields using regularized particle trajectories. *Inf. Process. Med. Imaging Proc. Conf.* **19**, 52–63 (2005).
12. Barrière, D. A. *et al.* In vivo magnetic resonance imaging reveals the effect of gonadal hormones on morphological and functional brain sexual dimorphisms in adult sheep. *Psychoneuroendocrinology* **109**, 104387 (2019).
13. Barrière, D. A. *et al.* Structural and functional alterations in the retrosplenial cortex following neuropathic pain. *Pain* (2019) doi:10.1097/j.pain.0000000000001610.
14. Mensch, A., Varoquaux, G. & Thirion, B. Compressed online dictionary learning for fast resting-state fMRI decomposition. in *2016 IEEE 13th International Symposium on Biomedical Imaging (ISBI)* 1282–1285 (2016). doi:10.1109/ISBI.2016.7493501.
15. Lee, M. H. *et al.* Clustering of Resting State Networks. *PLOS ONE* **7**, e40370 (2012).
16. Raichle, M. E. The Restless Brain. *Brain Connect.* **1**, 3–12 (2011).
17. Liu, C. *et al.* Anatomical and functional investigation of the marmoset default mode network. *Nat. Commun.* **10**, 1975 (2019).
18. Atzil, S., Gao, W., Fradkin, I. & Barrett, L. F. Growing a social brain. *Nat. Hum. Behav.* **2**, 624–636 (2018).
19. Menon, V. 20 years of the default mode network: A review and synthesis. *Neuron* **111**, 2469–2487 (2023).
20. De Ridder, D., Smith, M. L. & Adhia, D. Chapter 4 - Autonomic nervous system and the triple network: an evolutionary perspective with clinical implications. in *Introduction to Quantitative EEG and Neurofeedback (Third Edition)* (eds. Chartier, D. R., Dellinger, M. B., Evans, J. R. & Budzynski, H. K.) 63–77 (Academic Press, 2023). doi:10.1016/B978-0-323-89827-0.00016-4.
21. Jumah, F. R. & Dossani, R. H. Neuroanatomy, Cingulate Cortex. in *StatPearls* (StatPearls Publishing, Treasure Island (FL), 2024).
22. Natali, A. L., Reddy, V. & Bordoni, B. Neuroanatomy, Corticospinal Cord Tract. in *StatPearls* (StatPearls Publishing, Treasure Island (FL), 2024).
23. George, K. & M Das, J. Neuroanatomy, Thalamocortical Radiations. in *StatPearls* (StatPearls Publishing, Treasure Island (FL), 2024).
24. Inferior Longitudinal Fasciculus - an overview | ScienceDirect Topics. https://www.sciencedirect.com/topics/neuroscience/inferior-longitudinal-fasciculus.
25. Pieterman, K. *et al.* Cerebello-cerebral connectivity in the developing brain. *Brain Struct. Funct.* **222**, 1625–1634 (2017).
