# Supplementary material for: Affiliative behaviours regulate allostasis development and shape biobehavioural trajectories in horses
Source: Nat Commun. 2026 Jan 13;17:47. doi: 10.1038/s41467-025-66729-1 (PMC12800209; doi:10.1038/s41467-025-66729-1)
Supplement: Supplementary file 2 — Description of Additional Supplementary Files [file 41467_2025_66729_MOESM2_ESM.pdf]

Description of Additional Supplementary Files

File Name: Supplementary Data 1

Description: This plain text file (Excel) provides variables sorted by time and group, used in the statistical model.
