# Supplementary material for: Affiliative behaviours regulate allostasis development and shape biobehavioural trajectories in horses
Source: Nat Commun. 2026 Jan 13;17:47. doi: 10.1038/s41467-025-66729-1 (PMC12800209; doi:10.1038/s41467-025-66729-1)
Supplement: Supplementary file 4 — Reporting Summary [file 41467_2025_66729_MOESM4_ESM.pdf]

nature portfolio

Corresponding author(s): David André Barrière, Mathilde Valenchon, Matthieu Keller

Last updated by author(s): Oct 17, 2025

Reporting Summary

Nature Portfolio wishes to improve the reproducibility of the work that we publish. This form provides structure for consistency and transparency in reporting. For further information on Nature Portfolio policies, see our Editorial Policies and the Editorial Policy Checklist.

Statistics

For all statistical analyses, confirm that the following items are present in the figure legend, table legend, main text, or Methods section.

n/a	Confirmed	
☐	☒	The exact sample size (n) for each experimental group/condition, given as a discrete number and unit of measurement
☐	☒	A statement on whether measurements were taken from distinct samples or whether the same sample was measured repeatedly
☐	☒	The statistical test(s) used AND whether they are one- or two-sided<br>*Only common tests should be described solely by name; describe more complex techniques in the Methods section.*
☐	☒	A description of all covariates tested
☐	☒	A description of any assumptions or corrections, such as tests of normality and adjustment for multiple comparisons
☐	☒	A full description of the statistical parameters including central tendency (e.g. means) or other basic estimates (e.g. regression coefficient) AND variation (e.g. standard deviation) or associated estimates of uncertainty (e.g. confidence intervals)
☐	☒	For null hypothesis testing, the test statistic (e.g. F, t, r) with confidence intervals, effect sizes, degrees of freedom and P value noted<br>*Give P values as exact values whenever suitable.*
☒	☐	For Bayesian analysis, information on the choice of priors and Markov chain Monte Carlo settings
☒	☐	For hierarchical and complex designs, identification of the appropriate level for tests and full reporting of outcomes
☐	☒	Estimates of effect sizes (e.g. Cohen's d, Pearson's r), indicating how they were calculated

Our web collection on statistics for biologists contains articles on many of the points above.

Software and code

Policy information about availability of computer code

Data collection	All raw data collected for this article have been uploaded and are freely available : https://zenodo.org/records/13969827
Data analysis	All codes used for both statistical and MRI analysis in this article have been uploaded and are freely available : https://zenodo.org/records/13969827 and https://zenodo.org/records/10731031 and https://github.com/DavidBarriere/Equisobrain

For manuscripts utilizing custom algorithms or software that are central to the research but not yet described in published literature, software must be made available to editors and reviewers. We strongly encourage code deposition in a community repository (e.g. GitHub). See the Nature Portfolio guidelines for submitting code & software for further information.

Data

Policy information about availability of data

All manuscripts must include a data availability statement. This statement should provide the following information, where applicable:

- Accession codes, unique identifiers, or web links for publicly available datasets
- A description of any restrictions on data availability
- For clinical datasets or third party data, please ensure that the statement adheres to our policy

The whole dataset, along with the detailed statistical analyses, have been deposited on Zenodo (https://doi.org/10.5281/zenodo.13969827). The Turone Equine Brain templates and atlas toolkit has also been deposited on Zenodo and is publicly available at (https://doi.org/10.5281/zenodo.10731031).
These data can be shared and are publicly available without restriction.

Research involving human participants, their data, or biological material

Policy information about studies with human participants or human data. See also policy information about sex, gender (identity/presentation), and sexual orientation and race, ethnicity and racism.

Reporting on sex and gender	*Use the terms sex (biological attribute) and gender (shaped by social and cultural circumstances) carefully in order to avoid confusing both terms. Indicate if findings apply to only one sex or gender; describe whether sex and gender were considered in study design; whether sex and/or gender was determined based on self-reporting or assigned and methods used. Provide in the source data disaggregated sex and gender data, where this information has been collected, and if consent has been obtained for sharing of individual-level data; provide overall numbers in this Reporting Summary. Please state if this information has not been collected. Report sex- and gender-based analyses where performed, justify reasons for lack of sex- and gender-based analysis.*
Reporting on race, ethnicity, or other socially relevant groupings	*Please specify the socially constructed or socially relevant categorization variable(s) used in your manuscript and explain why they were used. Please note that such variables should not be used as proxies for other socially constructed/relevant variables (for example, race or ethnicity should not be used as a proxy for socioeconomic status). Provide clear definitions of the relevant terms used, how they were provided (by the participants/respondents, the researchers, or third parties), and the method(s) used to classify people into the different categories (e.g. self-report, census or administrative data, social media data, etc.) Please provide details about how you controlled for confounding variables in your analyses.*
Population characteristics	*Describe the covariate-relevant population characteristics of the human research participants (e.g. age, genotypic information, past and current diagnosis and treatment categories). If you filled out the behavioural & social sciences study design questions and have nothing to add here, write "See above."*
Recruitment	*Describe how participants were recruited. Outline any potential self-selection bias or other biases that may be present and how these are likely to impact results.*
Ethics oversight	*Identify the organization(s) that approved the study protocol.*

Note that full information on the approval of the study protocol must also be provided in the manuscript.

Field-specific reporting

Please select the one below that is the best fit for your research. If you are not sure, read the appropriate sections before making your selection.

[x] Life sciences [ ] Behavioural & social sciences [ ] Ecological, evolutionary & environmental sciences

For a reference copy of the document with all sections, see nature.com/documents/nr-reporting-summary-flat.pdf

Life sciences study design

All studies must disclose on these points even when the disclosure is negative.

Sample size	24 animals. The sample sizes were determined by the total number of foals available during the study year. Considering the ethical and logistical constraints associated with experimental work in large animals, all available individuals were included to ensure robust analyses while minimizing animal use. The sample sizes were reviewed and approved by the ethics committee.
Data exclusions	1 animals due to medical reason
Replication	Template and atlas of equine brain template have been created to ensure replication of the normalization step by peers.<br>statistical analysis code are available (https://zenodo.org/records/13969827) and ultimately for replication of the results by peers.<br><br>For plasma metabolites (total cholesterol and triglycerides) and IGF-1, measurements were performed in duplicate and the inter-assay and intra-assay coefficients of variation were <8%.<br>For ocytocin, measurements were performed in duplicate and the inter-assay and intra-assay coefficients of variation was 5.8 % at 40pg/mL and assay sensitivity was 7.8pg/mL.<br>For cortisol, measurements were performed in duplicate and the inter-assay and intra-assay coefficients of variation were 2.8% and 2.7% at 15ng/mL and 60ng/mL respectively. The assay sensitivity was 2ng/mL.<br>Finally, regarding behavioral results, further studies should be conducted to ensure replication of our study.
Randomization	Randomization procedure is explained in material and method section of the manuscript (line 367 - 386) :<br>"During the first five months of life, foals were all kept with their mothers in the same outdoor pasture. In October 2020, two distinct herds were formed and kept in two different pastures: 12 dyads of male foals/mothers (Herd A), and 12 dyads of female foals/mothers (Herd B). The protocol then started from November 2020 (Fig 1a). All procedures were performed in accordance with the European directive 2010/63/EU for animal protection and welfare used for scientific purposes and approved by the local ethical committee for animal experimentation (CEEA372VdL, Tours, France, ref. APAFIS #32985-2021091516064302). “With mother” and “without mother” treatments Half of the subjects were assigned to the “maternal presence” treatment (N=12: 6 males and 6 females) and half of them to the “maternal absence” treatment (N=12: 6 males and 6 females). The “maternal presence” and “maternal absence” foals were balanced according to date of birth, weight and paternal origins. The “maternal absence” treatment consisted in removing half of the mothers in each group (A and B) at the same time when the foals aged 6.64 ± 0.07 months old (mean ± SE). Consequently, after maternal separation, each herd (A and B) was composed of 6 without-

mothers foals, 6 with-mothers foals and their 6 adult mothers. Therefore, presence or absence of the mother was the sole difference between with or without mothers' foals. Foals have been kept within the same social herd (A or B) and could therefore interact with each other and with the adult mothers still present. One female foal belonging to the "with mother" leave the experiment 4-months after the maternal separation procedure due to medical reasons. Imaging, behavioural and physiological data from this animal have been retrieved for the final analysis."

Blinding

Laboratory and neuroimaging analyses were performed by a blinded experimenter. Blinding was not possible during behavioural observations due to the visible presence of the mares, but quantitative and standardized protocols were used to prevent bias.

Reporting for specific materials, systems and methods

We require information from authors about some types of materials, experimental systems and methods used in many studies. Here, indicate whether each material, system or method listed is relevant to your study. If you are not sure if a list item applies to your research, read the appropriate section before selecting a response.

Materials & experimental systems

n/a	Involved in the study	
☒	☐	Antibodies
☒	☐	Eukaryotic cell lines
☒	☐	Palaeontology and archaeology
☐	☒	Animals and other organisms
☒	☐	Clinical data
☒	☐	Dual use research of concern
☒	☐	Plants

Methods

n/a	Involved in the study	
☒	☐	ChIP-seq
☒	☐	Flow cytometry
☐	☒	MRI-based neuroimaging

Animals and other research organisms

Policy information about studies involving animals; ARRIVE guidelines recommended for reporting animal research, and Sex and Gender in Research

Laboratory animals

Subjects were 24 Welsh foals, 12 females and 12 males, aged 6.64 ± 0.07 months (mean ± SE) and their 24 mothers aged 8.2 ± 0.3 years on the day of the removal of the mothers. All animals (foals and mothers) were born and kept at the Animal Physiology Experimental Unit (UEPAO, INRAE, 37380 Nouzilly, France). They all lived in groups in, depending on the season, indoor collective stalls (20 m x 25 m) on straw with free access to an outdoor paddock (from November 26th, 2021 to April 23th, 2022), or in large outdoor grass pastures (April 23th, 2022 to June 27th, 2022). The dietary ration, primarily based on ad libitum access to forages, pasture and mineral block, was designed to meet the theoretical nutritional requirements (INRA 2011 tables) for weaned foals of this type and age51. Foals born between May 13th and June 27th, 2021. During the first five months of life, they were all kept with their mothers in the same outdoor pasture. In October 2020, two distinct herds were formed and kept in two different pastures: 12 dyads of male foals/mothers (Herd A), and 12 dyads of female foals/mothers (Herd B). The protocol then started from November 2020 (Fig 1a). All procedures were performed in accordance with the European directive 2010/63/EU for animal protection and welfare used for scientific purposes and approved by the local ethical committee for animal experimentation (CEEA VdL, Tours, France, ref. APAFIS #32985-2021091516064302).

Wild animals

Provide details on animals observed in or captured in the field; report species and age where possible. Describe how animals were caught and transported and what happened to captive animals after the study (if killed, explain why and describe method; if released, say where and when) OR state that the study did not involve wild animals.

Reporting on sex

Both sexes were included in the study, with balanced numbers of males and females in each group (50/50). However, the effect of sex could not be statistically tested independently, as sex was confounded with housing conditions: males and females had to be housed separately for ethical reasons to avoid reproduction risk among young animals. Sex and group information are nevertheless indicated in the figures for transparency.

Field-collected samples

For laboratory work with field-collected samples, describe all relevant parameters such as housing, maintenance, temperature, photoperiod and end-of-experiment protocol OR state that the study did not involve samples collected from the field.

Ethics oversight

All procedures were performed in accordance with the European directive 2010/63/EU for animal protection and welfare used for scientific purposes and approved by the local ethical committee for animal experimentation (CEEA372VdL, Tours, France, ref. APAFIS #32985-2021091516064302).

Note that full information on the approval of the study protocol must also be provided in the manuscript.

Plants

Seed stocks

Report on the source of all seed stocks or other plant material used. If applicable, state the seed stock centre and catalogue number. If plant specimens were collected from the field, describe the collection location, date and sampling procedures.

Novel plant genotypes

Describe the methods by which all novel plant genotypes were produced. This includes those generated by transgenic approaches, gene editing, chemical/radiation-based mutagenesis and hybridization. For transgenic lines, describe the transformation method, the number of independent lines analyzed and the generation upon which experiments were performed. For gene-edited lines, describe the editor used, the endogenous sequence targeted for editing, the targeting guide RNA sequence (if applicable) and how the editor was applied.

Authentication

Describe any authentication procedures for each seed stock used or novel genotype generated. Describe any experiments used to assess the effect of a mutation and, where applicable, how potential secondary effects (e.g. second site T-DNA insertions, mosiacism, off-target gene editing) were examined.

Magnetic resonance imaging

Experimental design

Design type

resting state

Design specifications

1 MRI session/animal, 10 min of functional resting state/session

Behavioral performance measures

Animals were anesthetized (ketamine/xylazine). functional MRI were performed first during MRI scanning session to limit long term effect of drugs. Both cardiac and respiratory rates were recorded to ensure similar anesthesia deepness between animals.

Acquisition

Imaging type(s)

functional, structural, diffusion

Field strength

3 Tesla

Sequence & imaging parameters

- for structural imaging, a three dimensional T1w MPRAGE acquired in coronal plane was used with the following parameters: Echo Time/Repetition Time=2.67ms/2500ms, Flip Angle=12°, Inversion Time=900ms, Number of Excitation=3, Partial Fourier=1, Slice Thickness=1 mm, Slice Number=208, Field of View=256x256mm, matrix=256x256, final resolution 1mm3.
- for diffusion imaging, we used a diffusion weighted MRI protocol based on a two dimensional T2w spin-echo sequence acquired in the axial plane, over 3 different shells optimized for Neurite Orientation Dispersion and Density Imaging (NODDI) model using the following parameters: Shell 1: b=300 s/mm2, 6 directions; Shell 2: b=700 s/mm2, 30 directions, and Shell 3: b=2000 s/mm2, 64 directions. The fixed parameters are Echo Time/Repetition Time=109ms/11.5s, Flip Angle=90°, Number of Excitation=1, Partial Fourier=0.75, Slice Thickness=2.4mm, Slice Number=57, Field of View =256x256mm, matrix=128x128, final resolution 2x2x2.4mm3, one b=0 per shell). Sequences have been acquired in different reading phases (left-right and right-left) for distortion corrections.
- for brain functional imaging a T2w spin-echo-planar imaging (SE-EPI) sequence acquired in the axial plane and left/right reading phase was used with the following parameters: Echo Time/Repetition Time=24ms/3.97s, Flip Angle=90°, Number of Excitation=1, Partial Fourier=1, Slice Thickness=3.3mm, Slice Number=40, Field of View=220x220mm, matrix=110x110, final resolution 2x2x3.3mm3, Number of repetition=250. Additionally, two similar sequences in different reading phases (left-right and right-left) of ten volumes each were acquired for distortion corrections.

Only one case of myositis has been detected after the imaging procedure, nevertheless the animal fully recovered within a week. DICOM data were converted to NIFTI format and organized as standardized data sets according to the Brain Imaging Data Structure (BIDS) using BIDScoin and are downloadable on Zenodo.

Area of acquisition

Whole brain scan

Diffusion MRI ☒ Used ☐ Not used

Parameters

for diffusion imaging, we used a diffusion weighted MRI protocol based on a two dimensional T2w spin-echo sequence acquired in the axial plane, over 3 different shells optimized for Neurite Orientation Dispersion and Density Imaging (NODDI) model using the following parameters: Shell 1: b=300 s/mm2, 6 directions; Shell 2: b=700 s/mm2, 30 directions, and Shell 3: b=2000 s/mm2, 64 directions. The fixed parameters are Echo Time/Repetition Time=109ms/11.5s, Flip Angle=90°, Number of Excitation=1, Partial Fourier=0.75, Slice Thickness=2.4mm, Slice Number=57, Field of View =256x256mm, matrix=128x128, final resolution 2x2x2.4mm3, one b=0 per shell). Sequences have been acquired in different reading phases (left-right and right-left) for distortion corrections.

Preprocessing

Preprocessing software

Softwares used for this project :
-BIDSCoin
- Ants
- FSL
- SPM8 (run on matlab 2018b)

- Nilearn
- itksnap
- Ginkgo

Normalization

Diffusion imaging data analysis

Because of its practical implementation, the NODDI model has become very popular to map the tissue microstructure in vivo and ex vivo in both clinical and preclinical applications. The NODDI model relies on a biophysical model that separates the diffusion of water into three diffusive compartments (intra-neurite, extraneurite and CSF), which are supposed as non-exchanging, contributing to the global diffusion attenuation. The net diffusion signal attenuation (A) corresponds to the following linear combination A resulting from a linear combination of the individual signal attenuations associated with each compartment, including: 1/ the neurite compartment of water molecules trapped within axons and dendrites characterized by a volume fraction (fic), 2/ the extra-neurite compartment characterized by a volume fraction (fec) and 3/ the CSF compartment containing free molecules with an isotropic displacement probability characterized by a volume fraction (fiso). Hence, the net signal diffusion signal A corresponds to the following linear combination:

A = fic · a.ic + fec · a.ec + fiso · a.iso

We pre-processed raw multishell diffusion imaging data and mapped estimated each of the previously described fractions using Ginkgo. First multishell diffusion imaging data were corrected for magnetic susceptibility distribution (topup), motions and eddy current-induced distortions (eddy). Then mean of B0 data for each animal was calculated and resulting images were used to calculate a diffusion weighted template using by modelbuild. Resulting template was then normalized to the TEBTA template using antsRegistrationSyNQuick. Then Ginkgo was used for NODDI analysis (DwiMicrostructureField) and released 4 maps: the fractional anisotropy, the intraneuritic fraction, the orientation dispersion index, and the isotropic fraction. Both linear and nonlinear transformation calculated by modelbuild and antsRegistrationSyNQuick were applied once to each contrast for spatial normalisation of the data within the TEBTA space using antsApplyTransforms. Then, images were spatially smoothed with an isotropic Gaussian kernel by convolving a 4 mm full width at half maximum. To assess regional FA, ICF, ODI and isoF changes over the groups each contrast was compared using an unpaired Student t test proposed by SPM. A brain mask was used to constrain the analysis to brain. For each cluster, the significance of the peak voxel was set as $p<0.05$ (t-score=1.72, degree of freedom=21). The results are presented on an axial and sagittal brain slice series generated with Nilearn.

Functional imaging data analysis

rs-fMRI data were pre-processed as previously described24,25,45. Briefly, EPI images were corrected for slice timing (slicetimer), motions (antsMotionCorr) and susceptibility distribution (topup). Then mean of EPI data for each animal was calculated and resulting images were used to calculate a functional template using by modelbuild. Resulting template was then normalized to the TEBTA template using antsRegistrationSyNQuick. Then images were detrended, low- and high-pass filtered (0.01Hz – 0.01Hz) the effect of the six previously calculated motion parameters including translations and rotations, both WM and CSF and global signals, were removed from data through linear regression using Nilearn.

Fractional amplitude of low-frequency fluctuations analysis

The fractional amplitude of low-frequency fluctuation (0.01–0.08 Hz) values were computed using the previously processed 4D data the fALFF function from 1000 Functional Connectomes Project was used to reveal the temporal and regional changes in GM occurring in fALFF maps. Then fALFF maps were spatially normalized to the TEBTA template using both linear and nonlinear transformation calculated previously and applied once using antsApplyTransforms. To assess regional fALFF changes over the groups each contrast was compared using an unpaired Student t test proposed by SPM. A brain mask was used to constrain the analysis to the brain. For each cluster, the significance of the peak voxel was set as $p < 0.05$ (t-score=1.72, degree of freedom=21). The results are presented on an axial and sagittal brain slice series generated with Nilearn.

Identification of the equine Default Mode Network (DMN)

To elicit for the first time the DMN in equidae, we calculated the mean Pearson correlation coefficient between time-series of spontaneous variations of BOLD signal, extracted from the functional ROIs previously calculated by the dictionary learning approach we defined functionally homologous to regions involved in DMN and DMN-like described in humans in numerous species (aG, aCC/pFC and RSC). Using data extracted and a multiple regression analysis approach we demonstrate that spontaneous BOLD signal fluctuation in these regions is highly correlated. For each cluster, the significance of the peak voxel was set as $p<0.001$ (t-score=3.5272, degree of freedom=21, FDR corrected). The results are presented on an axial and sagittal brain slice series generated with Nilearn.

Normalization template

in vivo brain template and atlas didn't exist before this experimental study so we created our template space using the following an updated method previously developed in Barrière DA et al 2019 Nature Communication.

Template creation

T1w MPRAGE data acquired for each animal were noise and signal bias corrected using Ginkgo and N4BiasFieldCorrection respectively and coregistered to the Johnson et al template46 using antsRegistrationSyNQuick. Coregistrated data were used to segment each brain using SPM and the GM, WM and CSF priors provided by Johnson et al. to create the GM, WM and CSF probabilistic maps of each subject. Denoised and signal bias corrected images were used in parallel to create a study specific T1w template, using modelbuild, an optimized pipeline using antsMultivariateTemplateConstruction2, an unbiased template building method developed in ANTs package. Once both linear and non-linear transformations were calculated for each animal, we compiled all the transformations calculated for each image and applied them once to corrected images using antsApplyTransforms to limit interpolation effects. Resulting images have been used to create the TEBTA template by calculating the mean image of each normalized T1w using Ginkgo. Probabilistic maps (GM, WM, CSF) have been normalized using the previous linear and non-linear transformations and GM, WM and CSF priors have been created by calculating the mean image of each normalized map using Ginkgo. Both templates and priors will be used for spatial normalization and segmentation for VBM analysis (see Voxel Based Morphometry Analysis section). The complete procedure is described Supplementary Material.

TEBTA atlas creation

For the creation of the TEBTA atlas, the equine brain atlas provided by Johnson et al is used as a starting point. Firstly the Johnson' template is linearly and nonlinearly coregistrated within the TEBTA template using antsRegistrationSyNQuick. Then,

both linear and non-linear transformations were applied to each ROI of the Johnson' atlas using antsApplyTransforms. Each normalized ROI has been visually inspected to check boundaries and accuracy of registration. Then some regions of interest (ROIs) such as Corpus Callosum, arbor vitae, and ventricular systems have been updated/added using WM and CSF priors for a best fit to these ROIs to the TEBTA template. Eventually, additional subcortical ROIs such as septum, preoptic hypothalamic area, nucleus accumbens, striatum, etc. have been drawn and delimited manually using fsleyes and itksnap. To propose a valuable parcellation of the equine cortex, we implemented the methods previously used by Garin et al for the establishment of a functional atlas in lemur mouse53. Briefly, a multi-animal dictionary learning statistical analysis was performed with Nilearn (random_state = 0) on preprocessed rsfMR images (see Functional Imaging Analysis section)54. A mask excluding the WM, CSF and subcortical areas was used to restrict the dictionary learning analysis to cortical functional data. The study based on 60 sparse components was selected for the final analysis. Each bilateral component was split into two unilateral regions and labelled left or right. This led to a 3D functional atlas composed by a mosaic of 55 local functional regions that were named using itksnap. The name of each ROI was defined using the names of brain structures reported by Schmidt et al55, the AAL2 human brain atlas but also using their structural connectivity (see TEBTA fiber atlas creation section). The complete procedure is described supplementary material.

TEBTA fiber atlas creation: Structural connectivity of the equine brain

Cortical ROIs resulting from the dictionary learning segmentation of the equine cortex were used to identify the largest bundle tracts of white matter to help for the identification of ROI based on their structural connectivity. For the construction of the fibre tractogram, we used the analytical Q-ball reconstruction model and streamline regularized deterministic (SRD) tractography algorithm available in Ginkgo56 (see details in supplementary methods). From obtained populational tractogram, we selected the large bundles (length>150mm) using a 2 steps approach: 1/ bundles between each cortical ROIs have been selected by a ROI-to-ROI selection, labelled and merged. Then bundles between thalamus and each cortical ROI have been selected as well as bundles between cerebellum and each cortical ROIs. With this selection, we expected to find long cortico-cortical pathways (i.e. cingulum), thalamic projections such as the somatosensorial tract to identify the somatosensory areas and the cerebellocortical tract to identify the motor areas. To eliminate non-relevant fibres previously selected within the bundle tract was filtered by length (>150mm) and by tortuosity (<2σ of mean of tortuosity). 2/ bundles selected have been visually inspected and 7 large bundle tracts (cingulum, corticospinal tract, anterior/posterior/inferior thalamic radiations, inferior longitudinal tracts and cerebellocortical tract) have been identified on basis of morphology, position within the brain, location and by comparison of fibre atlases available in humans and NHP. Areas connected to these tracts have been named according to their structural connectivity, position and literature. The complete procedure is described supplementary material.

Noise and artifact removal	Please see previous description
Volume censoring	Please see previous description

Statistical modeling & inference

Model type and settings	two sample t test
Effect(s) tested	1 effect "with mother" > "without mother"

Specify type of analysis: ☐ Whole brain ☐ ROI-based ☒ Both

Anatomical location(s): *Describe how anatomical locations were determined (e.g. specify whether automated labeling algorithms or probabilistic atlases were used).*

Statistic type for inference (See Eklund et al. 2016)	voxel-wize
Correction	FDR

Models & analysis

n/a	Involved in the study
☐	☒ Functional and/or effective connectivity
☐	☐ Graph analysis
☐	☐ Multivariate modeling or predictive analysis

Functional and/or effective connectivity	*Report the measures of dependence used and the model details (e.g. Pearson correlation, partial correlation, mutual information).*
Graph analysis	*Report the dependent variable and connectivity measure, specifying weighted graph or binarized graph, subject- or group-level, and the global and/or node summaries used (e.g. clustering coefficient, efficiency, etc.).*
Multivariate modeling and predictive analysis	*Specify independent variables, features extraction and dimension reduction, model, training and evaluation metrics.*
